# The Cellular Immune Response to Rabies Vaccination: A Systematic Review

**DOI:** 10.3390/vaccines7030110

**Published:** 2019-09-11

**Authors:** Lisanne A. Overduin, Jacques J.M. van Dongen, Leonardus G. Visser

**Affiliations:** 1 Department of Immunohematology and Blood Transfusion, Leiden University Medical Center, 2333ZA Leiden, The Netherlands; J.J.M.van_Dongen@lumc.nl; 2 Department of Infectious Diseases, Leiden University Medical Center, 2333ZA Leiden, The Netherlands; L.G.Visser@lumc.nl

**Keywords:** rabies, vaccination, rabies vaccine, cellular immunity, B-lymphocytes, T-lymphocytes, immunological memory, systematic review

## Abstract

The effectiveness of rabies vaccines is conventionally determined by serological testing. In addition to this assessment of humoral immunity, cellular immunity could help assess effectiveness and protection through a broad range of parameters. Therefore, this study aimed to systematically review all literature on the kinetics and composition of the cellular immune response to rabies vaccination in humans. A total of 1360 studies were identified in an extensive literature search. Twenty studies were selected for inclusion. In a primary response, plasma cells are detectable from day 7 to day 14, peaking at day 10. Memory B-cells appear from day 10 up to at least day 28. After revaccination, natural killer (NK) cells are the first detectable cellular parameters. Further research is required to assess cellular parameters in relation to long-term (serological) immunity. This review was registered in the International Prospective Register of Systematic Reviews (PROSPERO) under registration number CRD42019134416.

## 1. Introduction

The highly neurotropic rabies virus (of the genus Lyssavirus) causes fatal encephalitis upon infection in unvaccinated humans. With the highest known case-fatality rate [1], rabies accounts for 59,000 human deaths per year [2]. These deaths are, however, preventable by adequate pre- and post-exposure prophylaxis. For both strategies, vaccination with a modern inactivated cell-culture or embryonated egg-based rabies vaccine (CCEERV) is recommended [3]. Both intramuscular and intradermal administration routes have been proven to be equally effective [4,5].

The effectiveness of the rabies vaccine is conventionally determined by measuring the serum titer of rabies virus neutralizing antibodies (RVNA). A titer of 0.5 IU/mL or higher indicates an adequate B-cell response and is generally considered to be protective [6]. In the field of vaccinology, these serological antibody assays are traditionally employed for assessing the immune responses to vaccines, whereas cellular immune responses have not been studied as extensively.

Nonetheless, insight into the cellular immune response might help to determine the effectiveness of a vaccination regimen, using different parameters. Both the primary and secondary cellular immune response might contain relevant information related to the duration and strength of the protection acquired after vaccination. Therefore, this review aimed to provide an overview of the currently known kinetics and composition of the cellular immune response to the rabies vaccine, describing both B- and T-cell responses. In addition, the relation between the serological and T- and B-cellular responses was studied.

## 2. Materials and Methods

### 2.1. Search Strategy

This review was conducted following the Preferred Reporting Items for Systematic reviews and Meta-Analyses checklist (PRISMA) [7]. The protocol for this review could be found in the International Prospective Register of Systematic Reviews (PROSPERO) under registration number CRD42019134416. 

To collect data on the cellular immune response to rabies vaccination, a thorough literature search was performed. We consulted the databases of MEDLINE, Embase, Web of Science, COCHRANE Library, and Academic Search Premier with the search strategy provided in Appendix A. The search strategy included components for ‘rabies vaccination’ and ’cellular immune response’. Unpublished meeting abstracts were included in this review if they met the inclusion criteria.

### 2.2. Inclusion and Exclusion Criteria and Outcomes of Interest

We included studies that used inactivated cell-culture or embryonated egg-based rabies vaccine (CCEERV) in vivo or in human cells in vitro. A cellular immunological outcome in a human population needed to be assessed. Reviews and meta-analyses with references that contained relevant information to our questions were included as well, to identify missing studies in our initial search.

A study was excluded if it had no human component and if it only assessed serological or other non-cellular outcomes. Case reports and studies of which no full English text could be retrieved were excluded as well. Furthermore, studies published before 1983 were excluded, avoiding the inclusion of nerve tissue-based vaccines. Moreover, relevant immunological outcomes could not appropriately be assessed earlier.

Primary endpoints included all outcomes for B-cell responses, including the kinetics of the (memory) B-cell response, the quantity of (memory) B-cells, mean day of peak response, and composition of the response. Secondary endpoints included T-cell response outcomes, including the kinetics of the T-cell response, the number of T-cells, mean day of peak response, and composition of the response.

### 2.3. Selection Process

Two reviewers (L.A.O. and L.G.V.) independently screened the literature search results on eligibility for inclusion in this review. In the case of exclusion, the reason was documented. Unanimously selected studies were included in this review. Unanimously rejected studies were excluded. In case of discordance between the reviewers, the studies were reassessed by both of the reviewers, and together they concluded definitive inclusion or exclusion. Citations from review articles found in the literature search were checked to ensure that no studies were missed in the initial search. Eligible studies found in such a review, not found individually in our literature search, were included in our analysis.

### 2.4. Data Collection Process

One author (L.A.O.) developed a data extraction sheet and extracted relevant details. We collected the following study data: author(s), year of publication, country, study design, the total duration of follow up, type of vaccine, type of antigen for stimulation, route of administration, primary or booster vaccination regimen, and any relevant cellular immunological outcomes. Two authors (L.G.V. and J.J.M.v.D.) reviewed these extracted details.

### 2.5. Risk of Bias Assessment

The risk of bias was assessed using the Cochrane tool (ROBINS-I) [8]. One of the authors (L.A.O.) assessed the bias risk for any included study. For each study, the risk of bias was reported with a severity score, as determined by the Cochrane tool. The overall risk of bias was classified as the highest bias classification in any domain for a particular study. This assessment was reviewed by two other authors (L.G.V. and J.J.M.v.D.). General publication bias might have occurred, given the limited availability of sufficiently specific tests for detecting cellular outcomes, possibly resulting in unpublished studies in which no associations were found.

## 3. Results

### 3.1. Characteristics of Included Studies

**Figure 1:**
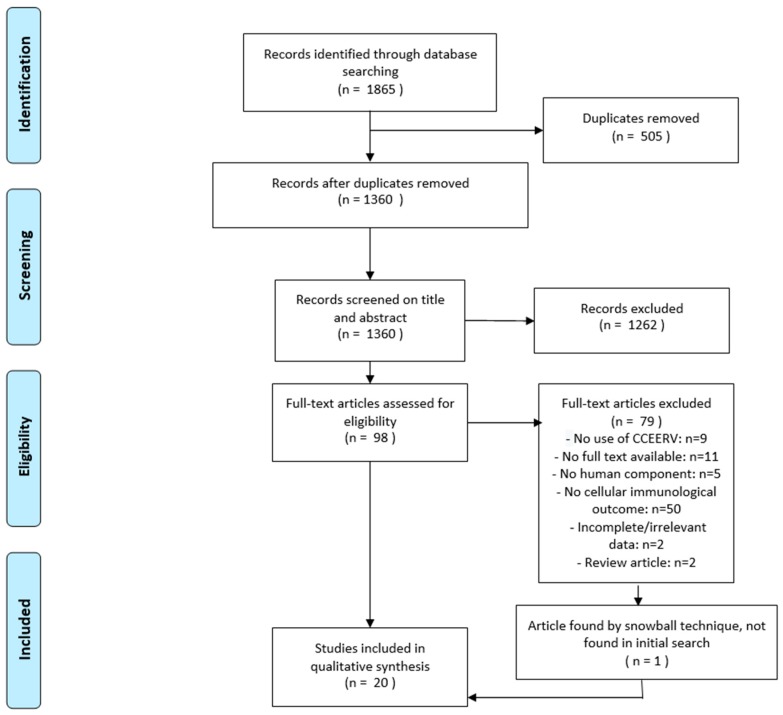
Flowchart describing the study selection process. CCEERV = cell-culture or embryonated egg-based rabies vaccines.

The literature search, which was performed on 17 April 2019, yielded 1811 results and 54 meeting abstracts (total = 1865), of which 1314 results and 46 meeting abstracts (total = 1360) remained after exclusion of duplicates. After screening, 1262 articles were excluded based on title and abstract. If available, the full text was retrieved for the remaining 98 articles. After evaluation of the full text, an additional 79 articles were excluded. One additional article (Ueki et al. 1990), which was not found in the initial search, was included. Thus, 20 articles were selected for final inclusion in this review (Figure 1). All of these studies were of prospective cohort design. The quality of all included studies was assessed and has been summarized with the characteristics of all studies in Table 1. 

### 3.2. B-Cell Responses

Serology is often used as a surrogate marker for the B-cell response. This could explain the scarcity of B-cell research, as only three studies that looked into cellular B-cell outcomes have been identified. 

The study of Ueki et al. described the kinetics of the B-cell response to an intradermally administered series of three vaccine doses (day 0, day 7, day 21). After a 4-week culture of Epstein-Barr virus (EBV)-infected B-cells, purified from peripheral blood mononuclear cells (PBMC) by selecting CD20^+^, CD5^+^, and CD5^-^ cells, rabies-specific B-cells were detected when challenged by disrupted rabies virus (ERA strain). Rabies-responsive (yet polyreactive) CD5^+^ IgM^+^-B-cells were detectable before primary vaccination. Seven days after the first dose, rabies-specific class-switched CD5^-^ IgG^+^- and IgA^+^-B-cells were detectable, accounting for the primary B-cell immune response. At day 14, seven days after the second dose, the IgM^+^-B-cell response increased twofold or threefold. But at day 7, after the third vaccination, IgM^+^-B-cell numbers had dropped back to prevaccination values.

**Figure 2:**
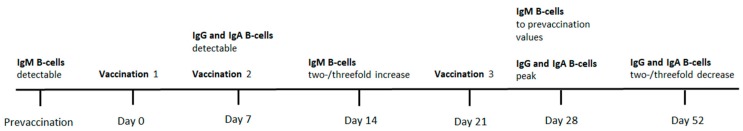
Timeline regarding the kinetics in Ueki et al. (1990).

Rabies-specific IgG+- and IgA+-B-cells did not change in numbers between day 7 to 14. The peak for IgG+- (11% of all IgG+ cells) and IgA+-B-cells (12.4% of all IgA+-cells) occurred at day 28, seven days after the third vaccination. Four weeks after the third vaccination, IgG+- and IgA+-B-cell numbers had decreased by half to two thirds (Figure 2). The height of their peak at day 28 could, however, be reproduced at day 163, after the fourth vaccination at day 142 [14]. 

The study by Van der Heijden et al. described that 11 weeks after revaccination in an immunized population, rabies-specific antibody-producing B-cells had a frequency of 1:10,000 among PBMC in an unstimulated, rabies-free in vitro environment. After 3 days of incubation with inactivated rabies virus, their frequency had increased to 1:150 [17].

The study by Blanchard-Rohner et al. compared the memory B-cell response to intramuscular revaccination to the primary B-cell response. The peak of the primary B-cell response was lower and later. After the first dose, IgG^+^-plasma cells could be detected from day 7 up to day 14, with a peak at day 10. After revaccination, this peak occurred as early as day 7, while the cells were detectable from day 4 to day 10. Memory B-cells were detectable at a constant level from day 10 to at least 28. The most rapid memory B-cell response occurred after the third dose in a three-dose pre-exposure regimen, being detectable even before this booster and rising from day 4 onward. The peak occurred at day 7.

Rabies-specific IgG antibodies could be detected and increased from day 10 after the first dose. Even higher IgG titers, than measured after the first vaccination, were detected from day 56, when a third vaccination would be administered. The IgG titers rose from day 7 after the third vaccination, coinciding with a plasma cell peak. Likewise, IgA antibodies rose from day 7 after the booster [24].

### 3.3. T-Cell Responses

Already in the early days, it was shown that the rabies vaccine is T-cell dependent. Addition of T-cells to non-T-cell peripheral blood lymphocytes (PBL), depleted of monocytes, in vitro was shown to promote maximal antibody formation. The combined T-cell and non-T-cell serological response was almost equal to the response of unseparated PBL [9,13].

Eleven studies assessed the cellular response to rabies vaccination, by using the lymphocyte proliferation assay. PBMC were exposed to various rabies antigens, followed by incubation with ^3^H-thymidine. The incorporation of the isotope is directly proportional to the level of proliferating lymphocytes and is expressed as the lymphocyte proliferation index (counts per minute in an antigen-enriched setting/counts per minute in an antigen-free setting). Depending on the study, different cut-off values were used to define a positive proliferative response (ranging from 1.3 to 3 and higher).

In almost all healthy individuals, in vitro proliferative responses occurred upon challenge with multiple different antigenic epitopes, such as the whole virus, the viral nucleocapsid, the ribonucleoprotein, and for different rabies virus strains [11,12,16,18]. For proliferative responders to the nucleocapsid (NC), it appeared that NC-specific cells outnumbered whole virus-specific cells (even if there was a whole virus-specific proliferative response). On the other hand, whole virus-specific cells outnumbered NC-specific cells in non-NC-responders. This shows that the most immunogenic epitopes differ between individuals [16].

Moore et al. reported a proliferative response in 6/10 vaccines on day 3 and 5/10 on day 7 after primary vaccination. In this study, a non-significant inverse association between the proliferation response and RVNA titer was found [22]

Herzog et al. determined the lymphocyte proliferation response at various intervals during a series of three vaccinations (day 0, 7, and 21). They reported that at day 7 (after the first double dose of rabies vaccine), 5/21 individuals showed a proliferative response indicative of a primary response. At day 21, two weeks after the second dose, 19/22 individuals had a proliferation response, and at day 33, 5 days after the third dose, all individuals did. Seroconversion (defined as at least 1 U/mL) was not associated with the proliferative response, with 2/22 seroconverted at day 7 and 22/22 seroconverted at day 21 [15].

In another in vitro booster setting, all individuals had a positive proliferation index for one or multiple rabies antigens at day 7 of incubation [18]. After revaccination, the peak of the lymphocyte proliferation index was reported to be at day 8 of incubation with rabies vaccine antigen [19,22]

The lymphocyte proliferation response and interferon (IFN)-γ production were found to be higher in the presence of plasma from immunized donors, containing anti-rabies (probably predominantly IgG) antibodies. This effect was more pronounced in the presence of low concentrations of rabies antigen [10]. The proliferation response and IFN-γ production also increased when stimulated with increasing levels of different rabies antigens (vaccine, G protein, and NC protein) [11]. The majority of the T-cell response appeared to consist of CD4^+^ T-cells [11,13,22]. Compared to day 0 of incubation with rabies antigens, flow cytometry showed an increase of CD4^+^ cells after 7 days of incubation, and a decrease in CD8^+^ cells [18].

After revaccination, natural killer (NK) cells appeared to be responsible for the major part of early IFN-γ production, even more so than T-cells. IFN-γ production in NK-cells was detectable from 12 h onwards and peaked at 18 h after a third vaccination. On the other hand, T-cell-mediated IFN-γ production occurred primarily from day 5 onwards, with a peak at day 7 after vaccination. The second wave of NK-mediated IFN-γ production coincided with IFN-γ production by the T-cells. NK cells also aided the immune response by degranulating and releasing perforin in response to rabies antigen at an early stage (6 h after revaccination). From 12 h after revaccination, CD8^+^ T-cells represented the majority of the cytotoxic response. The NK response (IFN-γ production and degranulation) seemed to be dependent on the presence of interleukin (IL)-2 (already detectable from 6 h) produced by antigen-specific CD4^+^ cells [26]. Another study, however, showed that exogenous IL-2 had no additional value to endogenous IL-2 for lymphocyte proliferation in vitro [13].

Immune responses are regulated by regulatory T-cells (Tregs). In an unpublished study, the frequency of Tregs in immunized subjects has been shown to increase from day 0 to day 14 after revaccination. Absolute Treg frequencies were, however, higher in the group that—serologically—responded best to the vaccine, both at day 0 and day 14 [27].

### 3.4. Intramuscular and Intradermal Vaccination

Of the 20 studies used in this review, four studies used the intradermal (ID) administration route as intervention, whereas eight used the intramuscular (IM) route. One study only used PBMC from naïve volunteers for in vitro stimulation, without a vaccination intervention [21]. In eight studies, the route of administration was not reported or unknown.

Only one study used both IM and ID vaccination, which allowed a within-study comparison. This study showed that type 1 (IFN-γ) and type 2 (IL-4) cytokine responses did not differ between intramuscular and intradermal post-exposure vaccination, 7 days after the last vaccination in either schedule. In this study, a high RVNA titer was associated with high levels of IFN-γ and IL-4 [28].

### 3.5. Cellular Immune Response in the Immunocompromised

Four studies looked into the cellular immune response in an immunocompromised group, of which two studies compared these results to healthy volunteers. In congenital immunodeficiency (CID) patients, an adequate lymphocyte proliferation response was found in 2/5 (40%), whereas this was 100% in healthy volunteers, four weeks after the first vaccine dose [20]. In 22/27 Human Immunodeficiency Virus 1 (HIV1)-infected patients, CD4^+^ cell counts increased up to 365 days after vaccination, which could also be due to other causes than the rabies vaccine, such as the coinciding increased use of and compliance to antiretroviral therapy. CD4^+^ cell counts were not associated with seroconversion state [25].

In juvenile idiopathic arthritis (JIA) and multiple sclerosis (MS) patients, who received rabies vaccination before autologous stem cell transplantation (ASCT), the lymphocyte proliferation index did not change significantly after revaccination six months after ASCT. Before transplantation and after the first vaccination, 2/5 JIA patients and 3/7 MS patients had a proliferative response. After transplantation, 1/5 JIA patients and 1/9 MS patients had a proliferative response. As ASCT is considered to wipe out immunological memory, the response after transplantation can be classified as a primary response [23].

Elderly are generally considered to be an immunocompromised group. In a study by Gomez et al., PBMC of unvaccinated individuals were stimulated with rabies vaccine antigen in vitro, and IL-2 and IFN-γ concentrations were determined by ELISpot assays. It was shown that produced IL-2 concentrations were antigen dose-dependent and increased in the elderly to a similar level as in the young. After a 4-day incubation period with a low dose of Human Diploid Cell Vaccine (HDCV) rabies antigen, PBMC of elderly showed a higher IFN-γ production than the young controls. However, this production of IFN-γ reached a plateau, whereas PBMC’s of the young volunteers could still increase their IFN-γ production over a 7-day incubation period or when stimulated with higher antigen doses [21].

## 4. Discussion

This study provided an overview of the composition and kinetics of the primary and secondary T- and B-cell responses to rabies vaccine in humans. Over the past thirty-six years, the cellular response to rabies vaccine has not been studied extensively. Evidence does, however, point in the direction of an important role for B- and T-cellular immunity in response to rabies vaccination, in addition to the well-known serological parameters

The fact that the rabies vaccine is a neoantigen for almost all people creates the possibility to study the kinetics and dynamics of primary and secondary cellular immune responses to vaccination under different conditions in controlled research settings.

The primary response to the rabies vaccine was mediated by CD4^+^ T cells. A CD4^+^ T-cell subset is an essential group of cells for an adequate immune response against rabies, as was shown by the large numbers of cells in this subset responding to antigen stimulation. This lymphocyte proliferation response was already detectable from day 3 onwards. After incubation with rabies vaccine antigen, a peak in the proliferative lymphocyte response (mainly CD4^+^ T-cells) was reported on day 8.

B-lymphocyte subsets displayed a peak at different time points. Plasma cells peaked on day 10 after primary vaccination, with a detection window from day 7 to 14. Memory B-cells were detectable from day 10 up to at least day 28. These peaks occurred later than the T-cell response peaks, which is a logical consequence of the T-cell dependency of this response

When B-cell kinetics were compared to the antibody response kinetics, the plasma cell peak was observed on day 10 after primary vaccination, but an increase in antibody titers usually occurred later. This showed that B-cell quantities were indeed a different parameter, that might be associated with, but was not replaceable by serological outcomes.

When a booster vaccination was applied, NK cells were the first cells that could be detected. NK cells, which are responsible for early IFN-γ production after stimulation by IL-2 produced by antigen-specific T-cells, were detectable from 12 h after revaccination. Moreover, they played a role in the early cytotoxic response as the main perforin producers up to 12 h after revaccination. CD8^+^ cells did not seem to play a major role in the cellular immune response to the rabies vaccine, as their relative numbers even tend to decrease after vaccination. They were, however, responsible for the ‘late’ (from 12 h onwards) perforin cytotoxicity after revaccination.

On day 7 after revaccination, all studied individuals showed a positive proliferation response for one or multiple rabies vaccine antigens. This showed that antigen-specific T-cells could produce an adequate response when boosted.

For the B-cell response, it was shown that the plasma and memory B-cell response occurred in great magnitude and speed after booster vaccinations. Furthermore, IgG and IgA B-cells were detectable at much higher quantities after booster vaccinations than after primary vaccination. This goes to show that booster vaccinations enhance the B-cell immune response.

Cytokine responses appeared to increase with an increasing amount of added rabies antigen. High IL-4 and IFN-γ levels were significantly associated with high RVNA titers.

The presence of anti-rabies antibodies enhanced the T-cell response as well. However, another study showed a non-significant association between low lymphocyte proliferation indices and high RVNA titers. Information on this topic is thus still inconclusive.

We performed a systematic review of the extensive literature on cellular responses to the rabies vaccine. Multiple literature databases were used, and an exhaustive literature search was performed, ensuring that no relevant articles would be overlooked. The qualitative synthesis that resulted provides a general overview of the kinetics and dynamics of B- and T-cellular immune responses.

Several limitations, however, need to be discussed. First, the extracted studies were relatively dominated by older publications using less advanced immunological assays, which limited the translation of the findings into a more detailed understanding of rabies vaccine immunology. Secondly, the extracted studies were highly heterogeneous, using different vaccine regimens, administration routes, immunological assays, and endpoints, making comparing studies extremely difficult. Thirdly, most studies had a small study population. which hampered finding correlations between cellular responses and antibody levels. Furthermore, one should be careful when interpreting these single, small-scale studies. Finally, we used the rather broad outcome measure ‘cellular immune response’, which might have biased our review and resulted in articles being incorrectly excluded. We have tried to account for this by having two reviewers review all results of the literature search independently.

In conclusion, a general pattern in the rabies-specific T- and B-cell immune response could be identified, but the lack of homogeneity among the studies hindered a meta-analysis. In any case, this review shows that cellular parameters can be assessed detailed enough to discriminate between individuals and that they indeed do differ between individuals. Serological responders appear to be a highly heterogeneous group from a B- and T-cellular perspective. Individuals tend to respond to different antigens and have different proliferation and cytokine production profiles. Kinetic parameters that were used in the studies that we described provide a rationale for timepoints and outcome measures in further research.

Besides, the described rabies-specific kinetics provide grips for more rational, evidence-based vaccination schemes. As was shown, memory B-cells were detectable only from day 10 onwards. Therefore, revaccination at day 3 or 7 in an immunization schedule may not contribute as much as expected to the memory response and long-term protection, and another time point (for example day 10) could be a more logical choice when new vaccination regimens are implemented.

Although T-cell proliferative responses to rabies vaccine challenges have been described to a relatively greater extent, B-cell responses can still be described in more detail with new techniques. Many more cellular parameters could be used to assess the immunogenicity and predict the (long-term) protection for a vaccine. Future research could look into how NK cell, B-cell, T-cell, and antibody kinetics relate to each other in primary and secondary responses under specific conditions. If future studies can connect long-term (serological) immunity to cellular and humoral parameters, this could provide the field of rabies immunology and general vaccinology with individual prediction models. For now, there are many opportunities in this field that are still to be explored.

## Figures and Tables

**Table 1:** Summary of included studies.

Authors	Year	Country	Population	N.	Follow-Up	Vaccine Type	Primary or Secondary ^A^	ROA	Immunization Schedule	Challenging Antigen	Risk of Bias
Uytdehaag et al. [9]	1983	The Netherlands	Healthy, naïve	N/R	2 or 3 weeks after each vaccination.	DKCV	Secondary	IM	Day 0, 1mo, 6mo	DKCV	Low
				N/R	2 or 3 weeks after each vaccination.	DKCV	Secondary	IM	Day 0, 7, 21	DKCV	
Celis et al. [10]	1985	USA	Immunized	5	Incubation.	N/R	Secondary	N/R	N/R	PM	Moderate
Celis et al. [11]	1986	USA	Immunized (high RVNA titer)	5	Maximum 6 mo after last vaccination. Incubation.	HDCV	Secondary	N/R	N/R	PMV	Moderate
Celis et al. [12]	1988	USA	Healthy	5	7–10 days after last immunization.	HDCV	Secondary	N/R	PrEP (N/S)	PM, CVS, ERA, PV, North American bat (NAB; strain 6014)	Moderate
Bunschoten et al. [13]	1989	The Netherlands	Naïve	7	Incubation.	DKCV	Secondary	IM	Day 0, 7, 21	PM-DKCV and PMV	Moderate
Ueki et al. [14]	1990	USA	Healthy, naïve (30–52 years)	3	Day -1, 7, 14, 28, 52, 163.	HDCV	Both	ID	Day 0, 7, 21, 142	ERA	Moderate
				1	Day -1, 7, 14, 28, 52, 163.	HDCV	Both	ID	Day 0, 7, 21	ERA	
Herzog et al. [15]	1991	France	Healthy, post-exposure, naïve to the vaccine (19–48 years)	22	Day 7, 21, 28/35.	PMV	Both	N/R	2× on day 0, 1× on 7, 21	CVS, ERA	Moderate
Herzog et al. [16]	1992	France	Healthy, immunized	18	Day 21.	PMV	Secondary	N/R	2× on day 0, 1x on 7, 21	ERA, NC	Serious
			Healthy, immunized	18	1–36 mo after the last vaccination.	PMV	Secondary	N/R	Day 0, 7, 28 + booster each two years	ERA, NC	
Van der Heijden et al. [17]	1993	The Netherlands	Naïve	25	Before immunization and variable times.	DKCV	Secondary	N/R	3 vaccinations	N/R	Serious
Thraenhart et al. [18]	1994	Germany	Immunized	18	After last immunization (2–14 years earlier). Incubation. Cell counts on day 0 and 7.	HDCV or PCECV	Secondary	N/R	N/R	PCECV, Flury LEP, GP, NP, RNP	Moderate
			Naïve	18	N/A	N/A	Primary	N/A	N/A	PCECV, Flury LEP, GP, NP, RNP	
Ghaffari et al. [19]	2001	USA	Healthy, naïve (23–33 years)	14	Prior to vaccination, 4 weeks after last vaccination.	HDCV	Secondary	IM	Day 0, 7, 28	N/R	Low
Brinkman et al. [20]	2003	The Netherlands	Healthy (19–49 years)	18	Day 0, 7, 14, 28, 365 days after 1st vaccination. Day 0, 7, 14, 28 after each booster.	HDCV	Both	IM	Day 0, 3 mo	PM-DKCV	Moderate
			CID patients (4–13 years)	5	Day 0, 7, 14, 28, 365 days after 1st vaccination. Day 0, 7, 14, 28 after each booster.	HDCV	Both	IM	Day 0, 3 mo	PM-DKCV	
Gomez et al. [21]	2004	Austria	Healthy, naïve (<35 years)	7	Incubation. Cell counts on day 0 and 7.	N/A	Both	N/A	N/A	HDCV	Moderate
			Healthy, naïve (>60 years)	8	Incubation. Cell counts on day 0 and 7.	N/A	Both	N/A	N/A	HDCV	
Moore et al. [22]	2006	USA	Healthy, naïve	5	N/R	N/A	Primary	N/A	N/A	CVS	Moderate
			Healthy, immunized	10	After last immunization (5 mo to 19 years earlier).	HDCV or PCECV	Secondary	N/A	N/A	CVS	
Brinkman et al. [23]	2007	The Netherlands	JIA or SLE patients (4–15 years) undergoing ASCT	6	Day 0, 28 after each vaccination.	HDCV	Both	IM	Day of bone marrow harvest, 6 mo after ASCT	PM-DKCV	Moderate
			MS patients (23–50 years) undergoing ASCT	10	Day 0, 28 after each vaccination.	HDCV	Both	IM	Day of bone marrow harvest, 6 mo after ASCT	PM-DKCV	
			Healthy (19–49 years)	18	Day 0, 28 after each vaccination.	HDCV	Both	IM	Day 0, 3 mo	PM-DKCV	
Blanchard-Rohner et al. [24]	2009	UK	Healthy, naïve (18–50 years)	10	Before immunization, day 2, 4, 7, 10, 14, 28 after 1st and 3rd vaccination.	HDCV	Both	IM	Day 0, 28, 56	N/R	Moderate
			Healthy, immunized (18–50 years)	10	After a single booster.	HDCV	Secondary	IM	Day 0	N/R	
Sirikwin et al. [25]	2009	Thailand	HIV1 patients, naïve (>15 years)	27	Day 0, 3, 7, 14, 30, 90, 180, 365.	PCECV	Both	8-site ID	Day 0, 3, 7, 14, 30	N/R	Moderate
Horowitz et al. [26]	2010	UK	Healthy, naïve (26–31 years)	30	Day 0, 21.	HDCV	Both	IM	Day 0, 7, 21	Inactivated virus	Moderate
Vejpongsa et al. [27] *(unpublished)*	2011	Thailand	Immunized (20–55 years)	41	Day 0, 14.	PVRV	Secondary	2-site ID	Day 0	NR	Moderate
Venkataswamy et al. [28]	2015	India	Naïve (25–40 years)	10	N/A	N/A	Primary	N/A	N/A	HDCV	Low
			Healthy, immunized (27–58 years)	10	Day 7 after the last vaccination.	PCECV	Secondary	ID	Day 0, 7, 28	HDCV	
			Healthy, immunized (27–58 years)	20	Day 7 after the last vaccination.	PCECV	Secondary	ID	Day 0, 7, 28, 180, 183	HDCV	
			Post-exposure (10–45 years)	18	Day 7 after the last vaccination.	PCECV	Secondary	ID	Day 0, 3, 7, 28	HDCV	
			Post-exposure (10–45 years)	20	Day 7 after the last vaccination.	PCECV	Secondary	IM	Day 0, 3, 7, 14, 28	HDCV	

Abbreviations: N. = number of individuals included in study group. ROA = route of administration. N/R = not reported. DKCV = Dog Kidney Cell Vaccine. IM = intramuscular. Mo = month(s). PM = Pitman–Moore. RVNA = rabies virus neutralizing antibodies. HDCV = Human Diploid Cell Vaccine. PMV = Pittman-Moore strain cultivated in Vero cells. PrEP = Pre-Exposure Prophylaxis. N/S = not specified. CVS = challenge virus strain. PV = Pasteur strain. ID = intradermal. NC = nucleocapsid. PCECV = Purified Chicken Embryo Cell Vaccine. LEP = low egg passage strain. GP = glycoprotein. NP = N protein. RNP = ribonucleoprotein. N/A = not applicable. CID = congenital immunodeficiency. JIA = juvenile idiopathic arthritis. SLE = systemic lupus erythematosus. MS = multiple sclerosis. ASCT = Autologous Stem Cell Transplantation. USA = United States of America. UK = United Kingdom. HIV1 = Human Immunodeficiency Virus 1. PVRV = Purified Vero Rabies Vaccine. ^A^: A primary response is defined as the response to the antigen in (cells of) a naïve individual. After any previous encounter with the antigen, the cells or individual cannot be considered naïve anymore, and, therefore, these responses were defined as secondary or memory responses.

## Supplementary Materials

Supplementary File 1

## Appendix A

Search strategy for all different databases:PubMed:

(((“rabies vaccination”[tw] OR “Rabies Vaccines”[mesh] OR “rabies vaccine”[tw] OR “rabies vaccines”[tw] OR rabies vaccin*[tw] OR “Rabipur”[tw] OR “RabAvert”[tw] OR “IMOVAX”[tw] OR ((“Rabies”[mesh] OR “Rabies virus”[mesh] OR “rabies”[tw]) AND (“Vaccination”[mesh] OR “vaccination”[tw] OR “vaccinations”[tw] OR vaccinat*[tw] OR “Vaccines”[mesh] OR vaccin*[tw]))) AND (“B-Lymphocytes”[mesh] OR “B-cell”[tw] OR “B-cell”[tw] OR B-cell*[tw] OR “B-Lymphocyte”[tw] OR “B-Lymphocytes”[tw] OR B-Lymphocyt*[tw] OR “T-Lymphocytes”[mesh] OR “T-cell”[tw] OR “T-cell”[tw] OR T-cell*[tw] OR “T-Lymphocyte”[tw] OR “T-Lymphocytes”[tw] OR T-Lymphocyt*[tw] OR “immune response”[tw] OR immune respons*[tw] OR “immunological response”[tw] OR immunological respons*[tw] OR kinetic*[tw] OR “Adaptive Immunity”[Mesh] OR “Adaptive Immunity”[tw] OR “Antigen-Mediated Clonal Selection”[tw] OR “Active Immunity”[tw] OR “Vaccine Immunogenicity”[tw] OR “Cellular Immunity”[tw] OR “Humoral Immunity”[tw] OR “Immunologic Memory”[tw])) OR “Rabies Vaccines/immunology”[majr]) AND english[la] NOT (“Animals”[mesh] NOT “Humans”[mesh]) AND (“1978/01/01”[PDAT]: “3000/12/31”[PDAT]) NOT ((“Case Reports”[ptyp] OR “case report”[ti]) NOT (“Review”[ptyp] OR “Clinical Study”[ptyp]))

### Appendix A.1. Embase

((“rabies vaccination”.mp OR exp “Rabies Vaccine”/OR “rabies vaccine”.mp OR “rabies vaccines”.mp OR rabies vaccin*.mp OR “Rabipur”.mp OR “RabAvert”.mp OR “IMOVAX”.mp OR ((“Rabies”/OR “Rabies virus”/OR “rabies”.mp) AND (exp “Vaccination”/OR “vaccination”.mp OR “vaccinations”.mp OR vaccinat*.mp OR exp “Vaccine”/OR vaccin*.mp))) AND (exp “B Lymphocyte”/OR “B-cell”.mp OR “B-cell”.mp OR B-cell*.mp OR “B-Lymphocyte”.mp OR “B-Lymphocytes”.mp OR B-Lymphocyt*.mp OR exp “T Lymphocyte”/OR “T-cell”.mp OR “T-cell”.mp OR T-cell*.mp OR “T-Lymphocyte”.mp OR “T-Lymphocytes”.mp OR T-Lymphocyt*.mp OR exp “Immune Response”/OR “immune response”.mp OR “immune respons*”.mp OR “immunological response”.mp OR “immunological respons*”.mp OR kinetic*.mp OR exp “Adaptive Immunity”/OR “Adaptive Immunity”.mp OR “Antigen-Mediated Clonal Selection”.mp OR “Active Immunity”.mp OR “Vaccine Immunogenicity”.mp OR “Cellular Immunity”.mp OR “Humoral Immunity”.mp OR “Immunologic Memory”.mp)) AND english.la AND exp “Humans”/AND (1978 OR 1979 OR 198* OR 199* OR 200* OR 201*).yr NOT ((exp “Case Report”/OR “case report”.ti) NOT (exp “Review”/OR exp “Clinical Trial”/))

### Appendix A.2. Web of Science

(ti=(“rabies vaccination” OR “Rabies Vaccine” OR “rabies vaccine” OR “rabies vaccines” OR “rabies vaccin*” OR “Rabipur” OR “RabAvert” OR “IMOVAX” OR ((“Rabies” OR “Rabies virus” OR “rabies”) AND (“Vaccination” OR “vaccination” OR “vaccinations” OR vaccinat* OR “Vaccine” OR vaccin*))) AND ts=(“B Lymphocyte” OR “B-cell” OR “B-cell” OR B-cell* OR “B-Lymphocyte” OR “B-Lymphocytes” OR B-Lymphocyt* OR “T Lymphocyte” OR “T-cell” OR “T-cell” OR T-cell* OR “T-Lymphocyte” OR “T-Lymphocytes” OR T-Lymphocyt* OR “Immune Response” OR “immune response” OR “immune respons*” OR “immunological response” OR “immunological respons*” OR kinetic* OR “Adaptive Immunity” OR “Adaptive Immunity” OR “Antigen-Mediated Clonal Selection” OR “Active Immunity” OR “Vaccine Immunogenicity” OR “Cellular Immunity” OR “Humoral Immunity” OR “Immunologic Memory”)) AND la=english AND py=(1978 OR 1979 OR 1980 OR 1981 OR 1982 OR 1983 OR 1984 OR 1985 OR 1986 OR 1987 OR 1988 OR 1989 OR 1990 OR 1991 OR 1992 OR 1993 OR 1994 OR 1995 OR 1996 OR 1997 OR 1998 OR 1999 OR 2000 OR 2001 OR 2002 OR 2003 OR 2004 OR 2005 OR 2006 OR 2007 OR 2008 OR 2009 OR 2010 OR 2011 OR 2012 OR 2013 OR 2014 OR 2015 OR 2016 OR 2017 OR 2018 OR 2019) NOT TI=(“case report” NOT (“Review” OR “RCT” OR “Trial”)) NOT ti=(veterinary OR rabbit OR rabbits OR animal OR animals OR mouse OR mice OR rodent OR rodents OR rat OR rats OR pig OR pigs OR porcine OR horse* OR equine OR cow OR cows OR bovine OR goat OR goats OR sheep OR ovine OR canine OR dog OR dogs OR feline OR cat OR cats)

### Appendix A.3. OR

(ts=(“rabies vaccination” OR “Rabies Vaccine” OR “rabies vaccine” OR “rabies vaccines” OR “rabies vaccin*” OR “Rabipur” OR “RabAvert” OR “IMOVAX” OR ((“Rabies” OR “Rabies virus” OR “rabies”) AND (“Vaccination” OR “vaccination” OR “vaccinations” OR vaccinat* OR “Vaccine” OR vaccin*))) AND ti=(“B Lymphocyte” OR “B-cell” OR “B-cell” OR B-cell* OR “B-Lymphocyte” OR “B-Lymphocytes” OR B-Lymphocyt* OR “T Lymphocyte” OR “T-cell” OR “T-cell” OR T-cell* OR “T-Lymphocyte” OR “T-Lymphocytes” OR T-Lymphocyt* OR “Immune Response” OR “immune response” OR “immune respons*” OR “immunological response” OR “immunological respons*” OR kinetic* OR “Adaptive Immunity” OR “Adaptive Immunity” OR “Antigen-Mediated Clonal Selection” OR “Active Immunity” OR “Vaccine Immunogenicity” OR “Cellular Immunity” OR “Humoral Immunity” OR “Immunologic Memory”)) AND la=english AND py=(1978 OR 1979 OR 1980 OR 1981 OR 1982 OR 1983 OR 1984 OR 1985 OR 1986 OR 1987 OR 1988 OR 1989 OR 1990 OR 1991 OR 1992 OR 1993 OR 1994 OR 1995 OR 1996 OR 1997 OR 1998 OR 1999 OR 2000 OR 2001 OR 2002 OR 2003 OR 2004 OR 2005 OR 2006 OR 2007 OR 2008 OR 2009 OR 2010 OR 2011 OR 2012 OR 2013 OR 2014 OR 2015 OR 2016 OR 2017 OR 2018 OR 2019) NOT TI=(“case report” NOT (“Review” OR “RCT” OR “Trial”)) NOT ti = (veterinary OR rabbit OR rabbits OR animal OR animals OR mouse OR mice OR rodent OR rodents OR rat OR rats OR pig OR pigs OR porcine OR horse* OR equine OR cow OR cows OR bovine OR goat OR goats OR sheep OR ovine OR canine OR dog OR dogs OR feline OR cat OR cats)

### Appendix A.4. COCHRANE Library

((“rabies vaccination” OR “Rabies Vaccine” OR “rabies vaccine” OR “rabies vaccines” OR “rabies vaccin*” OR “Rabipur” OR “RabAvert” OR “IMOVAX” OR ((“Rabies” OR “Rabies virus” OR “rabies”) AND (“Vaccination” OR “vaccination” OR “vaccinations” OR vaccinat* OR “Vaccine” OR vaccin*))) AND (“B Lymphocyte” OR “B-cell” OR “B-cell” OR B-cell* OR “B-Lymphocyte” OR “B-Lymphocytes” OR B-Lymphocyt* OR “T Lymphocyte” OR “T-cell” OR “T-cell” OR T-cell* OR “T-Lymphocyte” OR “T-Lymphocytes” OR T-Lymphocyt* OR “Immune Response” OR “immune response” OR “immune respons*” OR “immunological response” OR “immunological respons*” OR kinetic* OR “Adaptive Immunity” OR “Adaptive Immunity” OR “Antigen-Mediated Clonal Selection” OR “Active Immunity” OR “Vaccine Immunogenicity” OR “Cellular Immunity” OR “Humoral Immunity” OR “Immunologic Memory”)):ti,ab,kw

AND la = english AND py=(1978 OR 1979 OR 1980 OR 1981 OR 1982 OR 1983 OR 1984 OR 1985 OR 1986 OR 1987 OR 1988 OR 1989 OR 1990 OR 1991 OR 1992 OR 1993 OR 1994 OR 1995 OR 1996 OR 1997 OR 1998 OR 1999 OR 2000 OR 2001 OR 2002 OR 2003 OR 2004 OR 2005 OR 2006 OR 2007 OR 2008 OR 2009 OR 2010 OR 2011 OR 2012 OR 2013 OR 2014 OR 2015 OR 2016 OR 2017 OR 2018 OR 2019) NOT TI=(“case report” NOT (“Review” OR “RCT” OR “Trial”)) NOT ti = (veterinary OR rabbit OR rabbits OR animal OR animals OR mouse OR mice OR rodent OR rodents OR rat OR rats OR pig OR pigs OR porcine OR horse* OR equine OR cow OR cows OR bovine OR goat OR goats OR sheep OR ovine OR canine OR dog OR dogs OR feline OR cat OR cats)

### Appendix A.5. Academic Search Premier

TI((“rabies vaccination” OR “Rabies Vaccine” OR “rabies vaccine” OR “rabies vaccines” OR “rabies vaccin*” OR “Rabipur” OR “RabAvert” OR “IMOVAX” OR ((“Rabies” OR “Rabies virus” OR “rabies”) AND (“Vaccination” OR “vaccination” OR “vaccinations” OR vaccinat* OR “Vaccine” OR vaccin*))) AND (“B Lymphocyte” OR “B-cell” OR “B-cell” OR B-cell* OR “B-Lymphocyte” OR “B-Lymphocytes” OR B-Lymphocyt* OR “T Lymphocyte” OR “T-cell” OR “T-cell” OR T-cell* OR “T-Lymphocyte” OR “T-Lymphocytes” OR T-Lymphocyt* OR “Immune Response” OR “immune response” OR “immune respons*” OR “immunological response” OR “immunological respons*” OR kinetic* OR “Adaptive Immunity” OR “Adaptive Immunity” OR “Antigen-Mediated Clonal Selection” OR “Active Immunity” OR “Vaccine Immunogenicity” OR “Cellular Immunity” OR “Humoral Immunity” OR “Immunologic Memory”)) OR KW((“rabies vaccination” OR “Rabies Vaccine” OR “rabies vaccine” OR “rabies vaccines” OR “rabies vaccin*” OR “Rabipur” OR “RabAvert” OR “IMOVAX” OR ((“Rabies” OR “Rabies virus” OR “rabies”) AND (“Vaccination” OR “vaccination” OR “vaccinations” OR vaccinat* OR “Vaccine” OR vaccin*))) AND (“B Lymphocyte” OR “B-cell” OR “B-cell” OR B-cell* OR “B-Lymphocyte” OR “B-Lymphocytes” OR B-Lymphocyt* OR “T Lymphocyte” OR “T-cell” OR “T-cell” OR T-cell* OR “T-Lymphocyte” OR “T-Lymphocytes” OR T-Lymphocyt* OR “Immune Response” OR “immune response” OR “immune respons*” OR “immunological response” OR “immunological respons*” OR kinetic* OR “Adaptive Immunity” OR “Adaptive Immunity” OR “Antigen-Mediated Clonal Selection” OR “Active Immunity” OR “Vaccine Immunogenicity” OR “Cellular Immunity” OR “Humoral Immunity” OR “Immunologic Memory”)) OR SU((“rabies vaccination” OR “Rabies Vaccine” OR “rabies vaccine” OR “rabies vaccines” OR “rabies vaccin*” OR “Rabipur” OR “RabAvert” OR “IMOVAX” OR ((“Rabies” OR “Rabies virus” OR “rabies”) AND (“Vaccination” OR “vaccination” OR “vaccinations” OR vaccinat* OR “Vaccine” OR vaccin*))) AND (“B Lymphocyte” OR “B-cell” OR “B-cell” OR B-cell* OR “B-Lymphocyte” OR “B-Lymphocytes” OR B-Lymphocyt* OR “T Lymphocyte” OR “T-cell” OR “T-cell” OR T-cell* OR “T-Lymphocyte” OR “T-Lymphocytes” OR T-Lymphocyt* OR “Immune Response” OR “immune response” OR “immune respons*” OR “immunological response” OR “immunological respons*” OR kinetic* OR “Adaptive Immunity” OR “Adaptive Immunity” OR “Antigen-Mediated Clonal Selection” OR “Active Immunity” OR “Vaccine Immunogenicity” OR “Cellular Immunity” OR “Humoral Immunity” OR “Immunologic Memory”))

AND la = english AND py = (1978 OR 1979 OR 1980 OR 1981 OR 1982 OR 1983 OR 1984 OR 1985 OR 1986 OR 1987 OR 1988 OR 1989 OR 1990 OR 1991 OR 1992 OR 1993 OR 1994 OR 1995 OR 1996 OR 1997 OR 1998 OR 1999 OR 2000 OR 2001 OR 2002 OR 2003 OR 2004 OR 2005 OR 2006 OR 2007 OR 2008 OR 2009 OR 2010 OR 2011 OR 2012 OR 2013 OR 2014 OR 2015 OR 2016 OR 2017 OR 2018 OR 2019)

NOT ti(veterinary OR rabbit OR rabbits OR animal OR animals OR mouse OR mice OR rodent OR rodents OR rat OR rats OR pig OR pigs OR porcine OR horse* OR equine OR cow OR cows OR bovine OR goat OR goats OR sheep OR ovine OR canine OR dog OR dogs OR feline OR cat OR cats)

## References

[1] Fooks A.R., Banyard A.C., Horton D.L., Johnson N., McElhinney L.M., Jackson A.C. (2014). Current status of rabies and prospects for elimination. Lancet.

[2] Hampson K., Coudeville L., Lembo T., Sambo M., Kieffer A., Attlan M., Barrat J., Blanton J.D., Briggs D.J., Cleaveland S. (2015). Estimating the Global Burden of Endemic Canine Rabies. PLoS Negl. Trop. Dis..

[3] WHO Rabies Vaccines: WHO Position Paper. https://apps.who.int/iris/bitstream/handle/10665/272371/WER9316.pdf?ua=1.

[4] Denis M., Knezevic I., Wilde H., Hemachudha T., Briggs D., Knopf L. (2018). An overview of the immunogenicity and effectiveness of current human rabies vaccines administered by intradermal route. Vaccines.

[5] Langedijk A.C., De Pijper C.A., Spijker R., Holman R., Grobusch M.P., Stijnis C. (2018). Rabies Antibody Response After Booster Immunization: A Systematic Review and Meta-analysis. Clin. Infect. Dis..

[6] WHO Rabies Working Group Report, SAGE Meeting of October 2017. http://www.who.int/immunization/sage/meetings/2017/october/1_Background_paper_WG_RABIES_final.pdf?ua=1.

[7] Moher D., Liberati A., Tetzlaff J., Altman D.G. (2009). Preferred Reporting Items for Systematic Reviews and Meta-Analyses: The PRISMA Statement. PLoS Med..

[8] Sterne J.A., Hernán M.A., Reeves B.C., Savović J., Berkman N.D., Viswanathan M., Henry D., Altman D.G., Ansari M.T., Boutron I. (2016). ROBINS-I: A tool for assessing risk of bias in non-randomised studies of interventions. BMJ.

[9] UytdeHaag F.G., Osterhaus A.D., Loggen H.G., Bakker R.H., A Van Asten J., Kreeftenberg J.G., Van Der Marel P., Van Steenis B. (1983). Induction of antigen-specific antibody response in human peripheral blood lymphocytes in vitro by a dog kidney cell vaccine against rabies virus (DKCV). J. Immunol..

[10] Celis E., Wiktor T.J., Dietzschold B., Koprowski H. (1985). Amplification of rabies virus-induced stimulation of human T-cell lines and clones by antigen-specific antibodies. J. Virol..

[11] Celis E., Miller R.W., Wiktor T.J., Dietzschold B., Koprowski H. (1986). Isolation and characterization of human T cell lines and clones reactive to rabies virus: antigen specificity and production of interferon-gamma. J. Immunol..

[12] Celis E., Ou D., Dietzschold B., Koprowski H. (1988). Recognition of rabies and rabies-related viruses by T cells derived from human vaccine recipients. J. Virol..

[13] Bunschoten H., Klapmuts R.J., Claassen I.J.T.M., Reyneveld S.D., Osterhaus A.D.M.E., UytdeHaag F.G.C.M. (1989). Rabies Virus-specific Human T Cell Clones Provide Help for an in vitro Antibody Response against Neutralizing Antibody-inducing Determinants of the Viral Glycoprotein. J. Gen. Virol..

[14] Ueki Y. (1990). Clonal analysis of a human antibody response. Quantitation of precursors of antibody-producing cells and generation and characterization of monoclonal IgM, IgG, and IgA to rabies virus. J. Exp. Med..

[15] Herzog M., Fritzell C., Lafage M., Hirose J.A.M., Scott-Algara D., Lafon M. (1991). T and B cell human responses to European bat lyssavirus after post-exposure rabies vaccination. Clin. Exp. Immunol..

[16] Herzog M., Lafage M., Montaño-Hirose J.A., Fritzell C., Scott-Algara D., Lafon M. (1992). Nucleocapsid specific T and B cell responses in humans after rabies vaccination. Virus Res..

[17] Van der Heijden R.W., Langedijk J.P., Groen J., UytdeHaag F.G., Meloen R.H., Osterhaus A.D. (1993). Structural and functional studies on a unique linear neutralizing antigenic site (G5) of the rabies virus glycoprotein. J. Gen. Virol..

[18] Thraenhart O., Kreuzfelder E., Hillebrandt M., Marcus I., Ramakrishnan K., Fu Z., Dietzschold B. (1994). Long-Term Humoral and Cellular Immunity after Vaccination with Cell Culture Rabies Vaccines in Man. Clin. Immunol. Immunopathol..

[19] Ghaffari G., Passalacqua D.J., Bender B.S., Briggs D.J., Goodenow M.M., Sleasman J.W. (2001). Human Lymphocyte Proliferation Responses following Primary Immunization with Rabies Vaccine as Neoantigen. Clin. Diagn. Lab. Immunol..

[20] Brinkman D.M.C., Der Zijde C.M.J.-V., Dam M.M.T., Vossen J.M., Osterhaus A.D.M.E., Kroon F.P., Van Tol M.J.D. (2003). Vaccination with rabies to study the humoral and cellular immune response to a T-cell dependent neoantigen in man. J. Clin. Immunol..

[21] Gomez I., Marx F., Gould E., Grubeck-Loebenstein B. (2004). T cells from elderly persons respond to neoantigenic stimulation with an unimpaired IL-2 production and an enhanced differentiation into effector cells. Exp. Gerontol..

[22] Moore S.M., Wilkerson M.J., Davis R.D., Wyatt C.R., Briggs D.J. (2006). Detection of Cellular Immunity to Rabies Antigens in Human Vaccinees. J. Clin. Immunol..

[23] Brinkman D.M.C., Der Zijde C.M.J.-V., Dam M.M.T., Boekhorst P.A.W.T., Cate R.T., Wulffraat N.M., Hintzen R.Q., Vossen J.M., Van Tol M.J.D. (2007). Resetting the Adaptive Immune System After Autologous Stem Cell Transplantation: Lessons from Responses to Vaccines. J. Clin. Immunol..

[24] Blanchard-Rohner G., Pulickal A.S., Der Zijde C.M.J.-V., Snape M.D., Pollard A.J. (2009). Appearance of peripheral blood plasma cells and memory B cells in a primary and secondary immune response in humans. Blood.

[25] Sirikwin S., Likanonsakul S., Waradejwinyoo S., Pattamadilok S., Kumperasart S., Chaovavanich A., Manatsathit S., Malerczyk C., Wasi C. (2009). Antibody response to an eight-site intradermal rabies vaccination in patients infected with Human Immunodeficiency Virus. Vaccines.

[26] Horowitz A., Behrens R.H., Okell L., Fooks A.R., Riley E.M. (2010). NK cells as effectors of acquired immune responses: Effector CD4+ T cell-dependent activation of NK cells following vaccination. J. Immunol..

[27] Vejpongsa P., Leelasinjaroen P., Sodsai P., Hirankarn N., Tantawichien T. Increasing CD4+CD25hiFoxP3+ regulatory T-cells in subjects after repeated booster doses of rabies vaccination. Proceedings of the 21st ECCMID/27th ICC.

[28] Venkataswamy M.M., Madhusudana S.N., Sanyal S.S., Taj S., Belludi A.Y., Mani R.S., Hazra N. (2015). Cellular immune response following pre-exposure and postexposure rabies vaccination by intradermal and intramuscular routes. Clin. Exp. Vaccine Res..

